# Transmissibility of *Mycobacterium pinnipedii* in a murine model

**DOI:** 10.3389/fcimb.2024.1328981

**Published:** 2024-03-28

**Authors:** María Jimena Marfil, Federico Carlos Blanco, María Alejandra Colombatti Olivieri, María Emilia Eirin, Martín José Zumárraga

**Affiliations:** ^1^ Cátedra de Enfermedades Infecciosas, Facultad de Ciencias Veterinarias, Universidad de Buenos Aires, Ciudad Autónoma d Buenos Aires, Argentina; ^2^ Instituto de Agrobiotecnología y Biología Molecular (IABIMO) UEDD CONICET-Instituto Nacional de Tecnología Agropecuaria, Argentina (INTA), Centro de Investigación en Cs. Veterinarias y Agronómicas (CICVyA)-CNIA, Hurlingham, Buenos Aires, Argentina

**Keywords:** *M. pinnipedii*, *M. bovis*, transmissibility, mice, experimental model

## Abstract

The causative agent of tuberculosis in pinnipeds is *Mycobacterium pinnipedii*, a member of the *Mycobacterium tuberculosis* complex (MTC). The natural hosts are pinnipeds; however, other non-marine mammals, including humans, can also be infected. The transmissibility of a pathogen is related to its virulence. The transmissibility of a *M. pinnipedii* strain (i.e., 1856) was investigated in a murine model and compared with that of two *Mycobacterium bovis* strains (i.e., 534 and 04-303) with different reported virulence. Non-inoculated mice (sentinels) were co-housed with intratracheally inoculated mice. Detailed inspection of mice to search for visible tuberculosis lesions in the lungs and spleen was performed, and bacillus viability at 30, 60, and 90 days post-inoculation (dpi) was assayed. A transmissibility of 100% was recorded at 30 dpi in sentinel mice co-housed with the inoculated mice from the *M. pinnipedii* and *M. bovis* 04-303 groups, as evidenced by the recovery of viable *M. pinnipedii* and *M. bovis* from the lungs of sentinel mice. Mice inoculated with *M. pinnipedii* (1856) and *M. bovis* (534) survived until euthanized, whereas five of the *M. bovis* 04-303-inoculated mice died at 17 dpi. This study constitutes the first report of the transmissibility of a *M. pinnipedii* strain in mice and confirms the utility of this experimental model to study virulence features such as the transmission of poorly characterized MTC species.

## 1 Introduction

*Mycobacterium pinnipedii*, described as a new taxon of the *Mycobacterium tuberculosis* complex (MTC) early in this century (Cousins et al., 2003), is the causative agent of tuberculosis in wild seals, as has been reported worldwide (Zumárraga et al., 1999; Cousins et al., 2003). *M. pinnipedii* has a wide host range and zoonotic potential (Moser et al., 2008). Despite this classification, it shows genetic and biochemical differences compared with other members of the MTC (Alito et al., 1999). Its antigenic composition is different from that of *Mycobacterium bovis* strains. Indeed, *M. pinnipedii* strains, similarly to *Mycobacterium tuberculosis*, do not produce the MPB70 and MPB83 antigens (Alito et al., 1999), which distinguishes them from other MTC species such as *M. bovis*.

The first case of tuberculosis reported and described in wild seals occurred in an Australian marine park (Cousins et al., 1990). *M. pinnipedii* caused infection in several non-marine mammals, including humans, in the context of zoo outbreaks and free-ranging transmission (Cousins et al., 2003; Kiers et al., 2008; Moser et al., 2008; Loeffler et al., 2014; Zmak et al., 2019; Macedo et al., 2020). Moreover, the contact of wild seals with the habitants from the South American coasts may have facilitated the zoonotic transmission of *M. pinnipedii* in the pre-Columbian era (Bos et al., 2014). In New Zealand, infection with *M. pinnipedii* in beef cattle has been documented; however, the lack of multiple cases within the herds suggests that cow-to-cow transmission is uncommon (Loeffler et al., 2014). The transmission of *M. pinnipedii* to humans could occur via direct contact with aerosols, mucosal secretions, feces, or urine of pinnipeds (Deepak et al., 2019). In Australia, Thompson et al. (1993) described a human case of tuberculosis in a sea lion trainer, presumably caused by direct contact with these pinnipeds. Macedo et al. (2020) reported the first confirmed case of a zoonotic transmission of *M. pinnipedii* involving a sea lion (*Zalophus californianus*) and its keeper in a Portuguese zoo (Macedo et al., 2020). On the other hand, ecotourism activities, such as diving or snorkeling with sea lions, represent a potential risk of the zoonotic transmission of *M. pinnipedii* (Fiorito et al., 2020). Moreover, Macedo et al. (2020) suggest that further studies should be performed in order to draw conclusions about the potential transmission of *M. pinnipedii* to humans, particularly due to the typical “sea lion kisses” given to children in zoo shows worldwide (Macedo et al., 2020).

In this work, to expand the knowledge of this MTC species, a murine intratracheal model was used to study the *in vivo* transmission capacity of a *M. pinnipedii* strain isolated from an *Arctocephalus australis* from the Buenos Aires Province to co-housed sentinel mice. In addition, the transmissibility of this strain was compared with that of two *M. bovis* strains with distinct virulence phenotypes.

## 2 Materials and methods

The transmissibility of *M. pinnipedii* strain 1856 was evaluated by comparing its transmission with that of two *M. bovis* strains (i.e., 534 and 04-303) with distinct virulent phenotypes. The transmission model consisted of sentinels co-housed with intratracheally infected mice.

*M. pinnipedii* strain 1856 was originally isolated in 1992 from an adult female South American fur seal (*A. australis*) found in Las Toninas town, Buenos Aires Province, Argentina. The animal was found in very poor physical condition and died 24 h after arrival at the rehabilitation center (Bernardelli et al., 1996). This isolate was molecularly characterized by Zumárraga et al. (1999) and Romano et al. (1995). *M. bovis* strain 534 was originally isolated in 1994 from a bovine in Santa Fe Province, Argentina. This strain showed a low-virulence phenotype profile in a murine model (Zumárraga et al., 2008; Aguilar León et al., 2009). *M. bovis* strain 04-303 was originally isolated in 2004 from a wild boar (*Sus scrofa*) from La Pampa Province, Argentina, and was found to be highly virulent in experimental animal models such as mice (Zumárraga et al., 2008; Aguilar León et al., 2009), guinea pigs, and bovines (Meikle et al., 2011).

The experimental design consisted of a mouse aerosol transmission model of active infection with *M. tuberculosis* (Marquina-Castillo et al., 2009). Strain cultures were performed in Middlebrook 7H9 (BD, Difco™, Franklin Lakes, NJ, USA) supplemented with 0.4% sodium pyruvate (Anedra Research S.A., Troncos del Talar, Tigre, Buenos Aires, Argentina) and 0.05% Tween 80. The cultures were kept in a stove with continuous shaking to avoid the formation of clumps. Prior to the inoculation and to disaggregate bacterial clumps, the inoculum was passed through a 25-gauge syringe needle.

**Figure 1:**
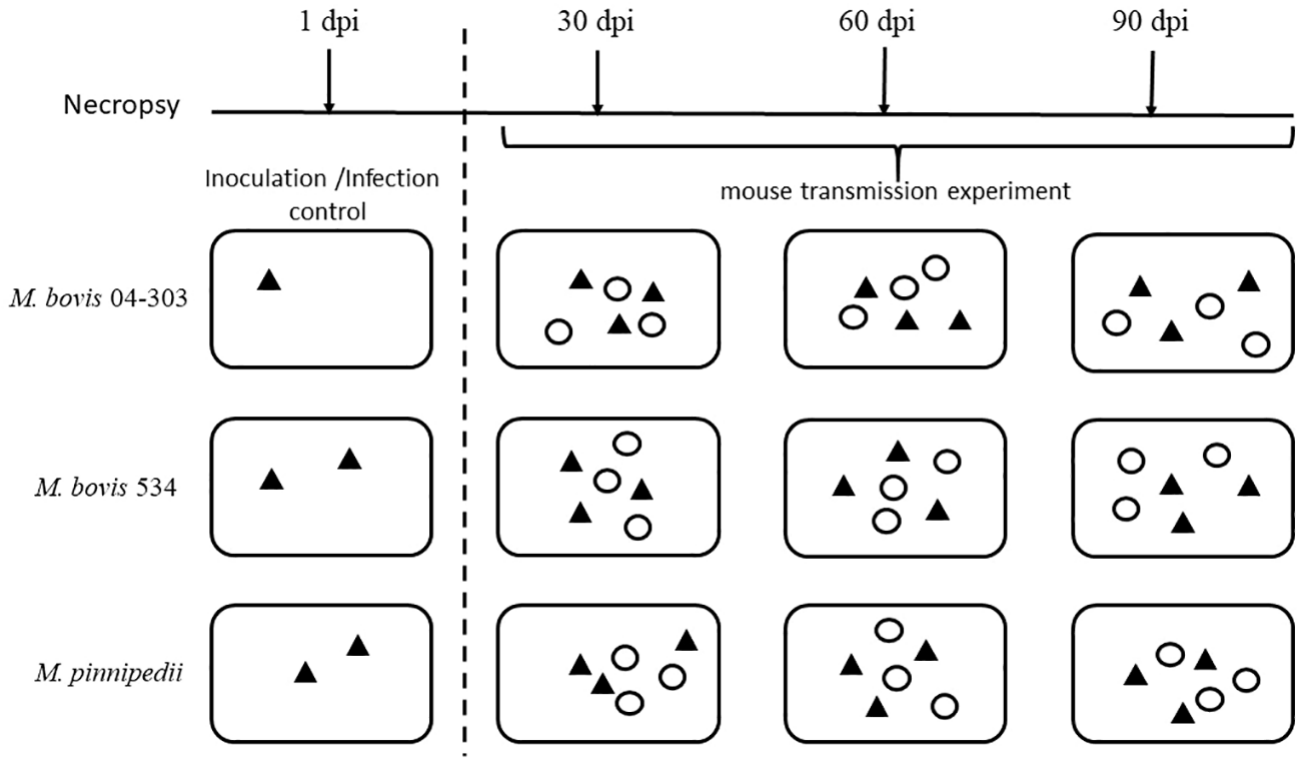
Experimental design. Animals were disposed in twelve ventilated cages. The first column shows three cages only had inoculated animals to confirm intratracheal infection. Then, columns 2-4 show cages evaluated in every evaluation time (30, 60 or 90 dpi), in which one inoculated and one sentinel mouse was randomly selected from each cage (three inoculated and three sentinel from each strain) for necropsy. Black triangles represent inoculated animals and white circles represents sentinels. dpi, days post inoculation.

A total of 64 female BALB/c mice (6–8 weeks old) were divided into three groups, with the group further classified into either “sentinels” or “inoculated.” To achieve infection, groups of 11 mice were inoculated with either the *M. pinnipedii* strain 1856 or the *M. bovis* strain 534. In addition, 10 mice were inoculated with the *M. bovis* strain 04-303. Of these mice, two from the *M. pinnipedii* 1856 and *M. bovis* 534 groups and one from the *M. bovis* 04-303 group were used as the controls for inoculation and infection and necropsied at 1 day post-inoculation (dpi). For the intratracheal inoculation, each mouse was sedated with isoflurane and then inoculated intratracheally as previously described (Aguilar León et al., 2009) with an inoculum of 2 × 10^5^ colony forming units (CFU)/100 µL of 1× phosphate-buffered saline (PBS) with each one of the evaluated *M. bovis* and *M. pinnipedii* strains. The remaining nine mice from each group (sentinels) were identified with a mark on the ear and distributed randomly into three cages containing three inoculated mice (infected) to establish a 1:1 infected/healthy ratio. Therefore, there were a total of six mice per cage (3:3). The groups of inoculated mice (and their sentinels) were housed in ventilated cages maintained under negative pressure. Food and water were available *ad libitum*. The mice were inspected daily for possible signs associated with tuberculosis, as previously described (Aguilar León et al., 2009), and survival was recorded up to 90 dpi. Necropsy was performed at 30, 60, and 90 dpi to search for visible tuberculosis lesions in the lungs and spleen. The experimental design is illustrated in **Figure 1**.

All procedures were performed in a class II type A2 biosafety cabinet in a biosafety facility. The five mice used as the inoculation and infection controls were necropsied at 1 dpi. The rest of the animals were processed as follows: three inoculated mice and three sentinel mice per group were necropsied. One inoculated mouse and one sentinel mouse were randomly selected from each of the three cages.

The organs were processed individually. Cross-contamination was avoided by using different sets of scissors and medical tweezers that were disinfected with sodium hypochlorite and subsequently cleaned with alcohol before reuse.

Assessment of the viability of bacilli in the organs consisted of the collection of the entire spleen and lungs in sterile collection tubes for aseptic homogenization in 3 mL of 1× PBS using a tissue homogenizer (Polytron, Kinematica, Luzern, Switzerland). Subsequently, a 100-µL aliquot of the homogenized organ was inoculated in Middlebrook 7H10 (BD, Difco™, Franklin Lakes, NJ, USA) supplemented with 0.4% sodium pyruvate (Anedra Research S.A., Troncos del Talar, Tigre, Buenos Aires, Argentina) in duplicate and incubated for 21–45 days at 37°C for the detection of mycobacterial growth.

As a transmissibility criterion, the strain that infected more sentinel animals in the shortest exposure time was considered the most transmissible.

The procedures described in the present study were approved by the Institutional Committee for the Care and Use of Experimental Animals of the Instituto Nacional de Tecnología Agropecuaria (CICUAE-INTA-CICVyA: 30/2014).

## 3 Results

### 3.1 Survival

Five mice inoculated with *M. bovis* strain 04-303 died at 17 dpi, while the other four mice in this group showed signs of consumption, tachypnea, and lethargy; therefore, these mice were euthanized according to the recommendations of CICUAE. As a result, there were no data available from this group at 30, 60, and 90 dpi. Mice inoculated with the *M. bovis* strain 534 and the *M. pinnipedii* strain 1856 were alive until the end of the experiment. Sentinel animals from all three groups were alive at the end of the transmission experiment (90 dpi), despite the variable virulence of the strains.

### 3.2 Infection

All of the animals used as the inoculation and infection controls yielded positive cultures from the lungs at 1 dpi (data not shown). Animals that were intratracheally inoculated with *M. pinnipedii* yielded isolates from all the spleen and lungs at the first evaluation period (30 dpi) (**Table 1**). In contrast, isolates were detected only in 33% (1/3) of the lungs of mice inoculated with *M. bovis* strain 534 at 30 dpi, but there were no isolates obtained from the spleen at this time point. Isolates from 55.6% (5/9) of the spleen and lungs of mice from the *M. bovis* 04-303 group were obtained at the first evaluation period (17 dpi) (**Table 1**).

**Table 1:** Proportion of animals with *M. pinnipedii* or *M. bovis* isolation in lungs and spleens.

		*M. pinnipedii* 1849		*M. bovis* 534		*M. bovis* 04-303	
		Lung (n/N)*	Spleen (n/N)	Lung (n/N)	Spleen (n/N)	Lung (n/N)	Spleen (n/N)
**30 dpi**	**I**	3/3	3/3	1/3	0/3	5/9**	5/9**
	**S**	3/3	1/3	1/3	0/3	3/3	0/3
**60 dpi**	**I**	2/3	ND	3/3	1/3	0/0	0/0
	**S**	3/3	3/3	2/3	0/3	3/3	0/3
**90 dpi**	**I**	ND	ND	1/3	0/3	0/0	0/0
	**S**	3/3	ND	3/3	0/3	3/3	2/3

*n/N represents the proportion between the number of animals from which viable M. pinnipedii or M. bovis strains were isolated and the total mice included in each group.

**Five animals died at 17 dpi and the remaining inoculated mice of the group (n=4) were euthanized.

I, Animals corresponding to the inoculated group; S, Animals corresponding to the sentinel (non-inoculated) group; ND, Not determined because of the contamination of the cultures.

Several of the tissue sample cultures from *M. pinnipedii*-infected mice became contaminated (i.e., with small, bright bacterial colonies and fungi), therefore resulting in less information on *M. pinnipedii* infection in inoculated animals at the later time points. A higher level of *M. bovis* 534 infection was detected at 60 dpi, with lung isolates from all three animals and with spleen isolates from 33% (1/3) of the animals. Fewer *M. bovis* 534 infections were detected at 90 dpi (i.e., 1/3 lungs and 0/3 spleen) (**Table 1**).

The transmission of *M. pinnipedii* strain 1856 and *M. bovis* strain 04-303 was efficient, with viable bacilli detected from the lungs of all sentinel mice at all three evaluation time points (**Table 1**). Conversely, the transmission of *M. bovis* strain 534 was progressive, with lung infection detected in one sentinel animal at 30 dpi, two at 60 dpi, and three at 90 dpi.

Infection in the spleen of sentinel mice in the *M. pinnipedii* group was progressive relative to the time points of the experiments. Bacilli were recovered from 33% (1/3) of the animals at 30 dpi and from 100% (3/3) at 60 dpi. Contamination prevented the identification of infection in the spleen at 90 dpi. *M. bovis* 04-303 was not detected in the spleen until 90 dpi, at which it was detected in 66% (2/3) of the sentinel animals. There was no spleen infection detected in *M. bovis* 534 sentinels (**Table 1**).

**Figure 2:**
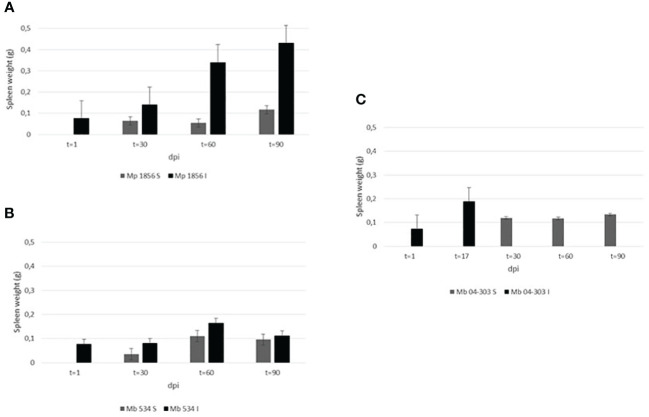
Comparison of spleen weights in the three groups among the inoculated and sentinel mice. Spleen of all animals were weighed at the end of the experiment or in those cases of *M. bovis* 04-303 inoculated mice where animals died. The weight was expressed in grams (g). Groups analyzed were as follows: **(A)** Mp 1856: mice inoculated with *M. pinnipedii* 1856 strain and sentinels, **(B)** Mb 534: mice inoculated with *M. bovis* 534 strain and sentinels, **(C)** Mb 04-303: mice inoculated with *M. bovis* 04-303 strain and sentinels. In all cases, comparison was performed between sentinel (non-inoculated) (S) and inoculated (I) animals, at different days’ post inoculation (dpi).

**Figure 3:**
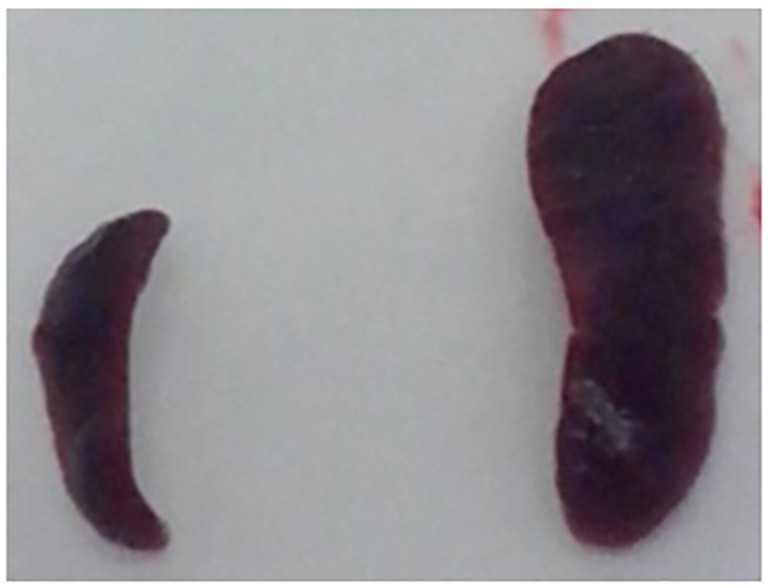
Comparison of the spleen size of sentinel and *M. pinnipedii* infected mouse. After necropsy, comparison *M. pinnipedii* inoculated and non-inoculated mice spleen size was performed. A marked difference in size was observed in the spleens from *M. pinnipedii* non-inoculated (left) and inoculated (right) mice.

**Figure 2** displays the comparison of the weights of the spleen from both inoculated and sentinel animals. The highest splenomegaly among the three groups of inoculated animals was observed in *M. bovis* 04-303 at 17 dpi, while that in the *M. bovis* 534-infected group was observed at 60 dpi, decreasing at 90 dpi (**Table 1**). On the other hand, a marked splenomegaly was observed in the spleen of *M. pinnipedii*-infected animals when compared with sentinel mice (**Figures 2**, **3**) at 60 and 90 dpi.

## 4 Discussion

The transmission capacity of pathogens is directly related to their virulence. Previously, we have demonstrated that the virulence level is correlated with a higher bacterial load, lung damage, and lower survival of infected BALB/c mice inoculated intratracheally with different *M. bovis* strains (Zumárraga et al., 2008; Aguilar León et al., 2009).

In this work, three different strains with variable virulence were studied. Two *M. bovi*s strains were used, named 534 and 04-303, as controls of the low- and high-virulence strains, respectively (Zumárraga et al., 2008; Aguilar León et al., 2009; Blanco et al., 2009; Meikle et al., 2011; Bigi et al., 2016; Marfil et al., 2016; Bigi et al., 2019). *M. bovis* strain 04-303 was previously described to be lethal at 21 dpi, causing sudden pneumonia with extensive necrosis lesions and high bacterial loads in the lungs and spleen of mice in a well-characterized mouse model of progressive pulmonary tuberculosis (Aguilar León et al., 2009). On the other hand, *M. bovis* strain 534 was observed to be less virulent, which allowed higher survival after 4 months of infection with limited tissue damage (Aguilar León et al., 2009). The authors concluded that this variability in the pathological damage and survival might be due to the induction of different patterns of immune responses during the infection (Aguilar León et al., 2009).

Marquina-Castillo et al., 2009 have demonstrated that transmissibility was related to the virulence phenotype in BALB/c mice inoculated intratracheally with different *M. tuberculosis* strains (Marquina-Castillo et al., 2009). Along these lines, Marfil et al. (2016) showed the high transmissibility of *M. bovis* 04-303 inoculated intratracheally (Marfil et al., 2016).

Another factor that could influence the infectivity of *M. bovis* is the inoculation route since this is relevant to the subsequent capacity of the bacteria to colonize the lung and, therefore, to replicate in this organ (Ferrara Muñiz et al., 2022). Ferrara Muñiz et al. (2022) evaluated the virulence of *M. bovis* (04-303) via subcutaneous inoculation and observed neither lesion nor death in mice inoculated with the hypervirulent *M. bovis* strain 04-303.

In the present study, *M. pinnipedii* was transmitted from intratracheally inoculated animals to all of the healthy sentinel animals at the first evaluated time point (30 dpi), thus showing the same transmission behavior as that of the hypervirulent *M. bovis* strain (i.e., 04-303) used as the control.

In spite of the variable virulence profiles of the strains used in the present study, the survival of all sentinel mice was not compromised, and although *M. pinnipedii* transmission occurred, the inoculum received was likely not sufficient to affect the survival of all mice at the evaluated time points.

Although these results are preliminary, all the lungs of the sentinel mice in the three groups were infected at the end of the transmission experiment (90 dpi). Similarly, all the lungs of the sentinel mice in the *M. pinnipedii* and *M. bovis* 04-303 groups were infected at 30, 60, and 90 dpi, indicating that these two strains are highly transmissible and that the transmission might have been through an airborne route. However, future experiments are necessary to confirm this conclusion as the digestive route, i.e., through the urine and feces, could also have occurred. On the other hand, *M. pinnipedii* colonized the spleen of mice faster or with a higher replication rate compared to *M. bovis* 04-303 and *M. bovis* 534. An evident splenomegaly was observed in mice inoculated with the *M. pinnipedii* strain, which reached a maximum at 90 dpi. However, this result could not be correlated with the respective bacteriological cultures as they were contaminated. However, at 60 dpi, in which a large splenomegaly was also evident, *M. pinnipedii* could be recovered by culture of all the spleen of the sentinel animals. Moreover, the splenomegaly in mice from the *M. bovis* 534-infected group progressively decreased from 60 to 90 dpi, showing bacterial clearance. This might be due to either antibody response or macrophage bactericidal capacity, as described by Altamura et al. (2001). However, only one *M. bovis* 534 spleen isolate was obtained at 60 dpi, and this was from an inoculated animal. There were no spleen isolates from the *M. bovis* 534 sentinels. The highest splenomegaly of *M. bovis* 04-303 at 17 dpi was related to the high virulence previously described for this strain. Bos et al. (2014) postulated that *M. pinnipedii* could have been the etiological agent responsible for the spread of tuberculosis among the inhabitants from the South American coasts in the pre-Columbian era. In this regard, the high transmissibility of *M. pinnipedii*, as suggested by these preliminary results, might be a good argument to explain the high rate of tuberculosis in the hunter-gatherer groups from the southernmost region of South America (Bastida et al., 2011).

Although the main hosts of *M. pinnipedii* are pinnipeds, this highly transmissible *Mycobacterium* species has a wide host diversity (Cousins et al., 2003; Kiers et al., 2008; Bastida et al., 2010; Loeffler et al., 2014; Macedo et al., 2020). However, the present study is the first report on the transmission of *M. pinnipedii* in a widely used experimental model to assess virulence and transmission, such as the intratracheal murine model.

Given the high transmissibility of *M. pinnipedii* demonstrated in the present study, special care should be taken in the management of this pathogen regarding mammalian infection in zoological parks and the conduct of recreational activities involving pinnipeds. Further experiments are necessary for a better understanding of host tropism and other aspects related to the intrinsic virulence of *M. pinnipedii* strains.

## Data availability statement

The original contributions presented in the study are included in the article/supplementary material. Further inquiries can be directed to the corresponding author.

## Ethics statement

The animal study was approved by The Institutional Committee for the Care and Use of Experimental Animals of the Instituto Nacional de Tecnología Agropecuaria (CICUAE-INTA-CICVyA: 30/2014). The study was conducted in accordance with the local legislation and institutional requirements.

## Author contributions

MM: Data curation, Formal analysis, Methodology, Writing – review & editing. FB: Methodology, Writing – review & editing. MC: Methodology, Writing – review & editing. ME: Writing – review & editing. MZ: Conceptualization, Formal analysis, Funding acquisition, Investigation, Project administration, Resources, Supervision, Writing – original draft, Writing – review & editing.
